# Remarks on the Curability of Inflammation

**Published:** 1875-12

**Authors:** L. A. Dugas

**Affiliations:** Augusta


### REMARKS ON THE CURABILITY OF INFLAMMATION.

By L. A. DUGAS, M.D., of Augusta.

*Read before the Library and Medical Association of Augusta, Ga.*

*What is inflammation?* If we look to Erichsen for a reply, we find that "the study of the inflammatory process is one of the most complex and difficult on which the surgeon can enter; but the labor required to master its details is well bestowed, inasmuch as an acquaintance with its nature, symptoms, and progress, gives an insight into a greater part of the science of surgery." He then adds: "Yet, as the discussion of this subject belongs rather to the domain of general pathology than to that of practical surgery, it cannot consistently be entered upon here." (P. 87, Am. ed., 1869.)

Turn to Gross, (p. 49, 1872,) and you find that "inflammation may be defined to be a perverted action of the capillary vessels of a part, attended with discoloration, pain, heat, swelling, and disordered function, with a tendency to effusion, deposits, or new products. In addition to these changes, there is also an altered condition of the blood and nervous fluid as an important element of the morbid process. In what inflammation essentially consists, it is as impossible to determine as it is to explain the intimate character of attraction, repulsion, gravitation, or cohesion."

Hear Ashhurst: "Authors, though differing as to the proper explanation to be given of many of the phenomena of inflammation, are, I think, generally agreed that those phenomena are mere modifications of the phenomena of natural textural life." (P. 34.)

It is not necessary to multiply references to authorities, for this would be simply to make confusion worse confounded. No attempt at a mere definition of inflammation can be successful. We must first recognize and study the fundamental physiological functions of the body; that is to say, innervation, capillary circulation, nutrition, and secretion, and we shall find that inflammation always involves a deviation from the normal condition of every one of these functions. While there may be lesions of either of these functions, or of several of them, without inflammation, this *always* exists when the whole are affected. And yet inflammations are not always alike; they differ infinitely, according to their cause and to the structure invaded. Inflammatory affections, so-called, are therefore as numerous and as various as the diseases classed under this head. No two are alike in their ramifications, nor in their history. They differ in causation, in symptoms, in duration, in terminations, and in curability. They are, therefore, strictly speaking, entities, and should be more generally admitted to be so. If this general assent could prevail we might dispense with the word inflammation, and allow each entity, or disease, to be judged and treated according to its own or individual peculiarities. But the word has been so long in use, and is so intimately interwoven with our facts, as well as theories, that we may not expect to see it set aside in our generation, nor probably in several more of them. With this explanation I will continue its use as a matter of convenience whenever it answers the purpose.

*Is inflammation curable?*—This may impress some as a ridiculous question, for it is in striking contrast with the dogmatic declarations of every period of the history of medicine. To intimate any doubt as to the efficacy of the modes of treatment advocated by leading men from Hippocrates to Broussais, would seem to be as preposterous in medicine as heresy is in theology. And yet the extravagancies of Broussais aroused a spirit of inquiry so potent that he lived to see his favorite dogmas, first doubted, and then almost unanimously discarded by the profession. His hobby, that, by depletion, all inflammations could be readily "removed," has now become obsolete, and every one knows that you may draw blood *ad deliquum animi* without causing a mere pimple upon the face, and that the same inefficiency attaches to any other form of antiphlogistic treatment. I wish it to be borne in mind that I use the term *cured* in its strict sense; that is to say, that the inflammatory process was then found to pursue the even tenor of its way in despite of any known form of treatment.

As it was my good fortune to be at the seat of war, whilst it progressed most furiously between the friends and adversaries of the Broussaisian school, I had abundant opportunities to verify the claims of the respective parties, and became satisfied that the victory was with the opposition, and that inflammation could not be *cured* by antiphlogistics, nor by any other plan of treatment then known. Whenever the inflammation was upon the surface, so as to be seen in its various stages, it was never arrested by treatment, but run its peculiar regular course to resolution, or to some other of its accustomed terminations. Such being the case with regard to inflammations subjected to ocular inspection, we could not reasonably suppose different results in the progress of inflammations affecting internal organs.

Such are the doctrines I continued to teach until about ten years ago; and the object of this paper is to lay before you how my views have undergone a change.

As far back as the introduction of quinine in the treatment of our malarial fevers, which were then considered inflammatory, and treated as such, I was forcibly impressed by the prompt and certain arrest of the disease by this wonderful agent. Could it be that quinine arrested inflammation? Or had we been in error with regard to the pathology of our fevers? The organs apparently implicated were out of sight, and we might have mistaken hyperæmia for inflammation. I became convinced that such was the fact, and the efficacy of quinine was to be found in its effects upon the nervous system, the blood, and the capillary circulation in general, thus bringing about an equalization of circulation and relief of *congestion*, not *inflammation*. There is no lack of evidence that by the timely use of quinine, we may *prevent* the development of inflammation in cases in which this would probably have occurred without it. We have also strong reasons for believing that quinine may modify the progress of inflammatory action after its occurrence; but I have yet to see a case, in which inflammation once set up, has been "jugulated" by quinine. It is true that quinine exerts a most beneficial effect upon pneumonia and dysentery, as they prevail in this section of the country, and that it is nearly our sheet anchor in these affections. But this is so because our forms of pneumonia and dysentery, especially when epidemic, are almost invariably mixed with malarial or paroxysmal fever, which readily yields to quinine, and leaves the inflammatory complication to subside more or less under judicious management.

About ten years ago I found that by applying the tincture of iodine to a feruncle the progress of inflammation was arrested, and was terminated by resolution. Repeated trials were attended with similar results. If applied even so late as the formation of the core, the pain would cease, the swelling would subside, and the core would come away in due time. You know that feruncles are of two kinds, the one simple and the other preceded by a vesicle, and therefore called the vesicular. From the resemblance of the latter form to carbuncles, I formerly designated them as *carbuncular feruncles*, but now prefer to call them *vesicular feruncles*, because this indicates their anatomical peculiarity. Moreover, carbuncles never begin with a vesicle. Vesicular feruncles are much more slow, painful and extensive than simple feruncles, and when they occur on the back of the neck they are frequently mistaken for carbuncles. If you will closely examine them in the initial stage, you will scarcely ever fail to detect a vesicle about the size of a pin head, attended with itching, and subsequently with tumefaction and pain. The vesicle is soon rubbed away by attempts to relieve the itching. The vesicle does not reappear, but the inflammation extends through the skin into the subjacent tissue, terminating in suppuration and sloughing.

Now if you apply the tincture of iodine to such cases, in any stage, you may safely expect to put a stop to any further inflammatory action. Both pain and tumefaction will readily subside, and the trying use of the knife be obviated.

I have never had an opportunity to test the efficacy of this treatment in genuine carbuncle, but am strongly disposed to think it might be equally beneficial as it is in feruncles.

But a much more useful application of this treatment is to be found in erysipelas, especially when consequent upon traumatic causes. The relief in such cases is sometimes so prompt as to partake of the marvellous. The last case I treated was that of a man who had sustained an injury to one of his fingers, and came to me with the whole hand greatly swollen, red and painful, with rapidly spreading erysipelas. I immediately gave the entire red surface a thorough painting with tincture of iodine, and advised its repetition in six and twelve hours. He visited me after the third painting, and I found the tumefaction much reduced, no pain, no tendency to spread, and every indication of the rapid subsidence of the disease. He was now advised to use the application only morning and night, and was well in a few days. This plan of treatment may be regarded as specific, when resorted to sufficiently early and in the proper way.

For feruncles and erysipelas, I usually direct the painting to be effectually done morning and night—in bad cases three times a day—and gradually discontinued as the disease disappears.

In the sub-cuticular and sub-cutaneous forms of whitlow, I have found the tincture of iodine sometimes beneficial, but not so in the thecal and periosteal varieties. For buboes, whether syphilitic or otherwise, I have no reason to think it perverts or lessens the tendency to suppuration. Indeed I am rather disposed to think I have seen suppuration oftener when it was used than when I resorted to other expedients. This may appear singular when we remember how valuable an agent the tincture is in dispersing some of the chronic enlargements of lymphatic glands.

We have endeavored to demonstrate:

1. That no *definition* of the word inflammation hitherto proposed is satisfactory.

2. That the so-called inflammation is a radical perturbation of the fundamental physiological functions, which varies according to its cause and the tissues involved.

3. That inflammations should be regarded and treated as entities or distinct diseases.

4. That at one time it was generally conceded that inflammatory affections were curable.

5. That the ultraism of Broussais instigated a spirit of inquiry, which resulted in the conviction that no treatment then known could be said strictly to cure or to arrest the regular progress of inflammatory action.

6. That this conviction became, with me, somewhat shaken by the introduction of quinine.

7. And finally, that it was demonstrated beyond doubt that some of the forms of inflammation may be effectually arrested by the application of tincture of iodine to the affected locality.

It has been wisely stated that there is nothing new under the sun, and I shall therefore not lay claims to originality which might be controverted by the more erudite; but I must say that my first use of tincture of iodine, as above narrated, was not instigated by knowledge derived from others. If others did the same before, I was not aware of the fact. I have ventured to place this paper at your disposal because I regard the discovery, by whomsoever made, of the fact that some forms of inflammation are undoubtedly curable, as one of the utmost importance. It not only corrects one of the convictions of the learned, but must lead to farther discoveries in the same direction. As one step in advance is only the precursor of others, let us redouble our exertions, in the hope that some one among us may have the honor of contributing the next fact.

