# Errata

**Published:** 1876-01

**Authors:** 


Errata.—The following errata occurred in the article of Dr. L. A. Dugas, published in our last number: 4th paragraph, 14th line, ramifications for manifestations; 5th paragraph, 12th line, causing for curing; 6th paragraph, 9th line, run for ran; 9th paragraph, 2d line from bottom, word *promptly* omitted after word *less;* feruncle for furuncle; 15th paragraph, 4th line, perverts for prevents.

